# Application of Hyperbranched Rolling Circle Amplification for Direct Detection of Mycobacterium Tuberculosis in Clinical Sputum Specimens

**DOI:** 10.1371/journal.pone.0064583

**Published:** 2013-06-04

**Authors:** Yang Liu, Yan-Ling Guo, Guang-Lu Jiang, Shi-Jie Zhou, Qi Sun, Xi Chen, Xiu-Jun Chang, Ai-Ying Xing, Feng-Jiao Du, Hong-Yan Jia, Zong-De Zhang

**Affiliations:** Laboratory of Molecular Biology for Tuberculosis, Beijing Tuberculosis and Thoracic Tumor Research Institute, Beijing Chest Hospital, Capital Medical University, Beijing, China; Institut de Pharmacologie et de Biologie Structurale, France

## Abstract

**Background:**

Global tuberculosis (TB) control is encumbered by the lack of a rapid and simple detection method for diagnosis, especially in low-resource areas. An isothermal amplification method, hyperbranched rolling circle amplification (HRCA), was optimized to detect *Mycobacterium tuberculosis* (*Mtb*) in clinical sputum specimens.

**Methods:**

A clinical validation study was performed to assess the diagnostic accuracy of HRCA. In order to analyze the detection limit of HRCA under optimal conditions, the method was initially used to detect purified H37Rv strain DNA and culture suspensions. Next, three strains of Mycobacterium tuberculosis complex (MTC) and eight strains of non-tuberculosis mycobacterium (NTM) were analyzed in order to evaluate specificity. Sputum specimens from 136 patients with diagnosed pulmonary TB, 38 lung cancer patients, and 34 healthy donors were tested by HRCA to validate the clinical application of HRCA for the rapid detection of *Mtb*.

**Results:**

The detection limit of HRCA for purified H37Rv DNA and culture suspensions was 740 aM and 200cfu/ml, respectively. The results of all MTC strains were positive in contrast to the NTM specimens which were all negative. The detection sensitivity for the 136 sputum specimens from TB patients was 77.2% (105/136), which was slightly lower than that of quantitative real-time PCR(79.4%, 108/136) and culture (80.9%,110/136). The sensitivity of all three methods was statistically higher than smear microscopy (44.9%, 61/136). The overall specificity of HRCA was 98.6% (71/72) which was similar to that of quantitative real-time PCR (qRT-PCR) and smear/culture methods (100%, 72/72).

**Conclusions:**

Use of the HRCA assay for detection of *Mtb* within clinical sputum specimens was demonstrated to be highly sensitive and specific. Moreover, the performance of HRCA is simple and cost-effective compared with qRT-PCR and is less time consuming than culture. Therefore, HRCA is a promising TB diagnostic tool that can be used routinely in low-resource clinical settings.

## Introduction

One-third of the world’s population is reportedly infected with mycobacterium tuberculosis (*Mtb*), the causative agent of pulmonary tuberculosis (PTB). The infectious disease kills nearly two million people annually and is still a serious public health problem in the world [1]. The lack of accurate, rapid, and cost-effective diagnostic tests poses a significant obstacle to TB control. A poorly functioning system for TB diagnosis ultimately leads to increased diagnostic delays and greater transmission of TB with high morbidity and mortality [2]. To date, routine TB laboratory diagnosis depends on smear microscopy and bacterial culture. Because of the low sensitivity of microscopy and the long time required for culture, feasible and accessible rapid diagnostic assays are urgently needed [3]. Recent development of commercially available tests, such as Gen-Probe Amplified Mtb Direct test (Gen-probe Inc., San Diego, California, USA), Roche Amplicor Mtb test (Roche Molecular Systems, Branchburg, New Jersey), Quantiferon In-Tube (Cellestwas, Melbourne, Victoria, Australia), and the TSPORT.TB (Oxford Immunotech, Oxford, UK),have improved our ability to detect *Mtb* [4]. However, the resources required for performing these tests remain above that of the ideal diagnostic test for use in developing countries, especially in low-resource clinical settings [2].

Hyperbranched rolling circle amplification (HRCA) with a padlock probe, an isothermal amplification method based on nucleic acid detection were developed and adapted for diagnostic purposes [5]. The padlock probe is a single-stranded linear oligonucleotide of −100 bases, with the sequences of the 5′- and 3′-ends complementary to the target sequence. When hybridized, head-to-tail, the ends of the probes are positioned adjacently and form a closed, circular molecule when incubated with a DNA ligase. The key point is that the ligation can only take place when both terminal segments recognize their target sequences correctly. Non-circularized probes are removed by exonuclease treatment, reducing subsequent ligation-independent amplification, while circularized probes may be amplified exponentially by HRCA with a pair of primers [6].

In this cohort study, we endeavored to validate the diagnostic accuracy of HRCA in our setting, the Beijing Chest Hospital, Capital Medical University, Beijing, China. This hospital is specialized for diagnosis and treatment of pulmonary diseases, especially TB and lung cancer. The padlock probe of HRCA, designed based on an insert sequence (IS6110) specific for MTC strains, was utilized to detect *Mtb.* The success of the HRCA detection method was compared with the results of laboratory-developed quantitative real-time PCR (qRT-PCR), fluorescence smear microscopy, and culture detection in sputum specimens from TB patients and a control group.

## Methods

### Mycobacteria Strains and Culture

Three strains of Mycobacterium tuberculosis complex (MTC) and eight strains of non-tuberculosis Mycobacterium (NTM) (Table 1) were analyzed by HRCA. All strains were supplied by China National Tuberculosis Reference Laboratory.

**Table 1:** Bacterial strains used in this study to determine the analytical specificity of HRCA.

Species	Reference strains^1^	HRCA result^2^
Mycobacterium tuberculosis complex (MTC)		
H37Rv	ATCC27294	p
M.*bovis*	ATCC19210	P
M.*africanum*	ATCC35711	P
Non-tuberculosis Mycobacterium (NTM)		
M. *avium*	ATCC 25291	N
M. *intracellulare*	ATCC 13950	N
M. *marinum*	ATCC 927	N
M.*kansasii*	ATCC 12478	N
M. *scrofulaceum*	ATCC 19981	N
M. *fortuitum*	ATCC 6841	N
M. *smegmatis*	ATCC 19420	N
M. *abscessus*	ATCC 19977	N

1 ATCC: American Type Culture Collection.

2 P: positive; N: negative.

Each strain was grown to mid-log phase in Middlebrook 7H9 liquid media (BD, USA) and stored frozen at −80°C. The number of colony forming units (CFU) for H37Rv bacterial stocks was pre-determined by bacterial enumeration of a ten-fold serial dilution cultured on Middlebrook 7H11 agar media (BD, USA).

### Study Population and Classification

From May 2010 to August 2012, sputum specimens were collected from 136 patients with diagnosed pulmonary TB at the Beijing Chest Hospital. The diagnosis of TB was based on clinical and radiological findings together with the positive identification of acid-fast bacilli (AFB) or culture. All patients were HIV seronegative. There were also 38 lung cancer inpatients from Beijing Chest Hospital and 34 healthy donors who had undergone physical examination in the hospital and who were designated healthy. Participants who could not provide an expectorated sputum specimen were excluded from the study. All work was approved by the institutional review boards of the Beijing Tuberculosis and Thoracic Tumor Research Institute. Informed consent was signed by everyone involved in the study.

All participants were classified into 4 groups depending on their clinical, microbiological, radiological evidences, and follow-up visits. The first group, designated as TB (S+/c+), consisted of patients with microbiologically confirmed pulmonary TB who tested positive by sputum smear (S+) and culture (C+) analyses.

The second group, designated as TB (S−/C+), consisted of patients with microbiologically confirmed pulmonary TB who were smear negative but *Mtb* L–J media culture positive (C+). The third group was designated as clinical TB(C−) based on clinical and radiological findings, and consisted of patients who were classified as having pulmonary TB, but lacking microbiological evidence of Mtb. All clinical TB(C−) patients showed a clear clinical response to TB treatment in the follow-up. The final group, designated as control group (Non-TB), consisted of lung cancer inpatients from Beijing Chest Hospital and healthy donors. All control group (Non-TB) participants were sputum smear- and culture-negative and no anti-TB treatments were given to the lung cancer inpatients.

### Sputum Sample Processing

Morning sputum specimens were digested and decontaminated by the NALC-NaOH, and then split into two aliquots. One was tested by HRCA and qRT-PCR while the other aliquot was processed for standard smear and culture. Fluorescence smear microscopy utilizing Auramine O stain and culture on Lowenstein-Jensen (L–J) media were performed as described previously [7] ^.^

QIAamp DNA Mini kit (Qiagen, USA) was used in tuberculosis genomic DNA extraction from sputum specimen. Firstly, Pipet 1 ml of digested sputa or bacterial culture into a 1.5 ml microcentrifuge tube, and centrifuge for 5 min at 5000 g. Remove the supernatant completely and discard; secondly, resuspend cell pellet in PBS to a final volume of 200 µl and boiled for 10 min, after cooled to the room temperature, Add 20 µl Qiagen Protease and 200 µl buffer AL to the sample, mix by pulse-vortexing for 30 s and incubate at 56°C for 10 min. The next DNA extraction procedures were performed according to manufacturer specifications. The extracted DNA was used for HRCA and qRT-PCR. DNA concentration was measured by the Nanodrop 2000 spectrophotometer (Thermo Scientific, USA) and then stored at −80°C until used in HRCA and real-time PCR.

### Padlock Probe Design

The sequence of IS6110, which was specific to the MTC, was selected as the target for the two ends of the padlock probe. To ensure the efficiency of padlock probe binding with the target DNA, the 5′- and 3′- end sequences were designed to bind adjacently, head-to-tail, with minimum secondary structure and complete complementary to the appropriate region of IS6110. The linker segment sequence was based on the sequence of the *Homo sapien* chromosome 12 genomic contig, which was not homologous to the IS 6110 sequence. In addition, primers, which were used to amplify the specific padlock probe signal during HRCA, were designed on the basis of the same region (Table 2).

**Table 2:** Sequences of the padlock probe and primers used in HRCA and qRT-PCR.

Name	sequence (5′–3′)
Padlock probe	GACTCGACACCCCAC *aatgagtcttgggaAGCCTCTG*
	*TATGTGTGTATGTGTATGTACAAACACATATATA*
	*tttagtcccctggctccAAGA* CAGCTGTGTGCAGATC
Prime HRCAa	AATGAGTCTTCTTGGGA
Prime HRCAb	GGAGCCAGGGGACTAAA
Prime PCR a	AGGGCGAACGCGATTTTA
Prime PCR b	CGGCTGATGTGCTCCTTG

The capital and underlined letters indicated the sequences complementary to IS 6110; the small italics and letters were the primer (Primer HRCA a and b) sequence used in HRCA for circular padlock probe amplification; Primer PCR a and b were used in qRT-PCR assay.

### Padlock Probe Ligation and Exonuclease Treatment

The DNA extracted from the sputum specimen or bacterial cultures were used in a 60 µl ligation reaction for the purpose of forming the circular padlock probe. 15 µl extracted DNA was incubated at 95°C for 10 min, and was then added to the 60 µl reaction mixture, which contained 3 µl ligase (Epicentre Biotechnologies, USA) at 100 U/ul, 6 µl 10× reaction mixture (Epicentre Biotechnologies, USA), 3 µl 50 mM MgCl_2_, 3 µl 1 mM ATP, 15 µl ss DNA probe at 10 uM and 15 µl sterile ddH_2_O, followed by incubation for 60 min at 60°C and inactivation of the ligase at 85°C for 10 min.

After ligation, 1 µl exonuclease I at 20 U/ul (Epicentre Biotechnologies, USA)was added and incubated for 90 min at 37°C, followed by inactivation at 85°C for 15 min.

### Hyperbranched Rolling Circle Amplification

The 50 µl HRCA reaction contained 5 µl padlock probe at 10 uM, 10 units φ29 DNA polymerase (New England Biolabs, UK), 5 µl 10× phi29 reaction mixture, 1 µl bovine serum albumin, 5 µl primers(Table 2) at 10 uM for padlock probe amplification, 2 µl 10 mM dNTPs and 31 ul sterile ddH_2_O. The circular padlock probe signals were amplified by incubation with gently shaking at 37°C for 1 h, followed by inactivation at 65°C for 10 min. After the reaction, 10 µl products were loaded on a 2% agarose gel and visualized under UV light. The positive signals were visualized as a ladder of bands with about 100bp intervals.

### Quantitative Real-time PCR

Quantitative real-time PCR (qRT-PCR) was performed as a control method using the My IQ™ iCycler machine (Bio-Rad, USA) and SsoFast EvaGreen Supermix kits (Bio-Rad, USA). The sequence of IS 6110 was amplified in a 20 µl volume containing 10 µl supermix, (Bio-Rad, USA) 1 µl 10 µM primers (Table 2), 1 µl extracted template DNA and 8 µl sterile ddH_2_O. The procedure for the real-time PCR was 2 min at 95°C, then followed by 95°C, 15 sec and 60°C, 60 sec, 40 cycles.

### Statistical Analysis

SPSS for Windows, version 17.0 (Statistical Package for Social Sciences, Chicago, IL, USA) was used for statistical analyses. *Mtb* culture positivity was used as a reference standard for identification of *Mtb*. So, in this study, culture method was used as control for evaluating the efficacy of the other three assays. Sensitivity, specificity, as well as positive and negative predictive values (PPV & NPV) was calculated for the HRCA and qRT-PCR in comparison with culture. The chi-square test was used to analyze the data. A P value of <0.05 was considered statistically significant.

## Results

### The Detection Limits of HRCA and qRT-PCR Under Optimal Conditions

**Figure 1:**
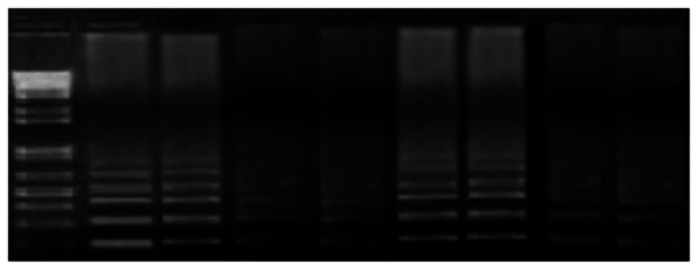
Detection limits of HRCA. Agarose gel analysis of HRCA products. Line1 (the left most) is 100 bp DNA marker. Line 2 to 9 (from left to right), 10 µ of the HRCA products were analyzed b 2% agarose gel electrophoresis followed by ethidium bromide staining. The specimen detected by HRCA: line 2 to line 5 is H37Rv purified DNA of 740 fM, 74 fM,7.4 fM, 740 aM; line 6 to 9 is H37Rv culture suspension of 2×10^5^ cfu/ml, 2×10^4^ cfu/ml, 2×10^3^ cfu/ml, 2×10^2 ^cfu/ml. Because no bands were seen in the 74 aM and 2×10^1 ^cfu/ml samples, the sensitivity of HRCA in detection of purified DNA and culture suspension is 740 aM and 2×10^2 ^cfu/ml, respectively.

**Figure 2:**
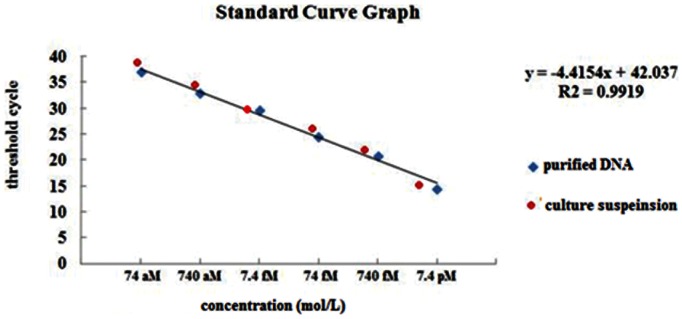
Detection limits of qRT-PCR. The specimen of H37Rv purified DNA, with concentrations of 740 fM, 74 fM, 7.4 fM, 740 aM, 74 aM, 7.4 aM and the H37Rv culture suspension,with concentrations of 2×10^5^ cfu/ml, 2×10^4^ cfu/ml, 2×10^3^ cfu/ml, 2×10^2 ^cfu/ml, 2×10^1^ cfu/ml, 2×10^0^ cfu/ml, were analyzed by qRT-PCR. No positive signals were detected by the end of 40 cycles in specimens of 7.4 aM and 2 cfu/ml. The sensitivity of qRT-PCR on purified DNA and culture suspensions is 74 aM and 2×10^1^cfu/ml, respectively.

In order to investigate the sensitivity of HRCA for purified DNA, which was extracted and purified from H37Rv culture using QIAamp DNA Mini Kit (Qiazen, USA), the purified DNA was diluted by a series of 1∶10 dilutions from 7.4 pM to 7.4 aM and then tested by HRCA and qRT-PCR. The sensitivity of the HRCA and qRT-PCR for purified DNA was 740 aM (Fig. 1) and 74 aM (Fig. 2), respectively.

For analysis the sensitivity of HRCA for strain culture suspension, the culture suspension of H37Rv was diluted by a series of 1∶10 dilutions from 2×10^6^ cfu/ml to 2 cfu/ml, then purified DNA was extracted from each of the diluted culture suspensions and tested by HRCA and qRT-PCR. The sensitivity of the HRCA and qRT-PCR for strain culture suspension was 200 cfu/ml (Fig. 1) and 20 cfu/ml (Fig. 2), respectively.

To analyze the specificity of HRCA for MTC, MTC strains including H37Rv, M.bovis, M. africanum, as well as eight strains of NTM (Table 1) were tested by HRCA. Only MTC strains could be detected by HRCA with the IS6110-designed padlock probe and no positive results for NTB detection were observed, which indicated perfectly high specificity of the HRCA assay.

### Classification of the Study Population and the Diagnostic Performance of HRCA or qRT-PCR Per Classification Group

For assessment of the clinical application of HRCA for direct detection of MTB in clinical sputum specimen, 136 pulmonary TB patients, 38 lung cancer patients, and 34 healthy donors were involved and sputum specimens were collected.

In 136 pulmonary TB patients, the number of positive cases detected by HRCA, qRT-PCR, smear microscopy, and culture was 105, 108, 61 and 110, respectively (Table 3). In the smear positive patients (S+/C+), 59 out of 61were positive by HRCA and qRT –PCR, representing a 96.7% sensitivity (95% CI 92.2% to 100%). The two assay-negative patients were later identified as positive for infection with M. *avium* and M.kansasii, respectively. In the group with smear negative and culture positive (S−/C+) participants, only 44 out of 49 patients were positive by HRCA assay, representing a sensitivity of 89.8% (95% CI, 81.3% to 98.3%). The sensitivity of qRT-PCR was 95.9% (95% CI, 90.4% to 100%) with 47 out of 49 patients testing positive,which is statistically higher than that of HRCA in this group (95.9% vs 89.8%, p = 0.009). Among the 26 patients in the group of clinical TB(C−), who had a clinical diagnosis of TB but no *Mtb*- positive sputum culture, HRCA and qRT-PCR detected two additional positive sputum specimens. Both of these patients had a history of previous TB diagnosis and treatment. The gel bands displayed by the HRCA assay for these specimens were very weak and the Ct-value from the qRT-PCR assay were very high (Ct 36–37), which showed a low amplification signal.

**Table 3:** Classification of participants with HRCA and qRT-PCR results for each group.

Group	% of total(n)	HRCA+	HRCA−	qRT-PCR+	Qrt-PCR−
		% in group(n)	% in group(n)	% in group(n)	% in group(n)
TB(S+/C+)	29.3(61)	96.7^a^(59)	3.3(2)	96.7^d^(59)	3.3(2)
TB(S−/C+)	23.6(49)	89.8^b^(44)	10.2(5)	95.9^e^(47)	4.1(2)
TB(C−)	12.5(26)	3.8(2)	96.2(24)	3.8(2)	96.2(24)
Non-TB	34.6(72)	1.4(1)	98.6^c^(71)	1.4(1)	98.6^f^(71)
Total	100(208)	50.5(106)	49.5(102)	51.9(109)	48.1(99)

a = sensitivity in this group, 95%CI = 92.2% to 100%.

b = sensitivity in this group, 95%CI = 81.3% to 98.3%.

c = specificity in this group, 95%CI = 96% to 100%.

d = sensitivity in this group, 95%CI = 92.2% to 100%.

e = sensitivity in this group, 95%CI = 90.4% to 100%.

f = specificity in this group, 95%CI = 96% to 100%.

### Comparative Performance of HRCA, qRT-PCR, Smear, and Culture Methods in All Participants Evaluated for TB Diagnosis

Culture assay was the “gold” standard method for identification of *Mtb* and used as control in this study in order to evaluate the efficacy of the other three assays. Overall, HRCA sensitivity was 77.2%(95% CI, 70.2% to 84.3%)(Table 4), which was slightly lower than that of culture assay sensitivity (80.9%) but did not show statistical difference (p = 0.344). However, HRCA sensitivity was significantly higher than that of smear microscopy (44.9%), which was statistical significant (p<0.01). The sensitivity of qRT-PCR was 79.4% (95% CI, 72.6% to 86. 2%) which was also slightly lower than that of culture assay sensitivity (80.9%, p = 1.00) and was significantly higher than that of smear microscopy (44.9%, p<0.01). Although in the TB(S−/C+) group the sensitivity of qRT-PCR was significantly higher than that of HRCA, no statistical difference was found between the sensitivity of qRT-PCR and HRCA (79.4% VS 77.2%, p = 0.25) when comparing all patient groups.

**Table 4:** TB diagnostic performance of smear microscopy, culture, HRCA, and qRT-PCR assays.

Assay	TB (n = 136)	Non-TB(n = 72)	SE (%)	SP (%)	PPV (%)	NPV (%)
Smear **+**	61	0	44.9	100	100	48.0
Smear −	75	72				
Culture**+**	110	0	80.9	100	100	73.5
Culture−	26	72				
HRCA**+**	105	1	77.2	98.6	99.1	69.6
HRCA−	31	71				
qRT-PCR**+**	108	1	79.4	98.6	99.1	71.8
qRT-PCR−	28	71				

SE: sensitivity, SP: specificity, PPV: positive predictive value, NPV: negative predictive value.

In the control group, among all 72 non-TB participants, one lung cancer patient was HRCA and qRT-PCR positive, representing a specificity of 98.6%, which was similar with that of smear and culture (100%). The corresponding assay-positive patient had a history of *Mtb* infection based on chest X-Ray evidence.

## Discussion

Tuberculosis is still a major global health problem and the limitations of conventional laboratory diagnoses are clear. Developing improved TB diagnostics is an international research priority and offers great promise in the fight against TB [8]–[9]. However, the cost of technological advancement remains a major obstacle to implementation of novel methods of TB detection in low-resource countries [2]. Therefore, a new diagnostic method should be evaluated for performance and feasibility of implementation in low-resource clinical settings.

In this study, HRCA with a padlock probe designed by the sequence of IS 6110, was developed and evaluated for efficacy in direct detection of *Mtb* from clinical sputum samples. Firstly, under optimal conditions, HRCA showed high sensitivity and reproducibility in detection of purified DNA (740 aM) and culture suspensions (200 CFU/ml) of H37Rv. Although the HRCA product was checked and visualized by electrophoresis with an agarose gel, the detection limit was an order of magnitude lower than that of qRT-PCR (74 aM or 20 CFU/ml) and other in-house PCR (50 CFU) [10] methods. The limit was also lower than that for Cobas TaqMan MTB PCR (Roche, Basel, Switzerland), a commercialized amplification method, with an assay sensitivity of 4.0 copies/ul [11] or 18 CFU/ml in sputum specimens [12]. Nevertheless, detection limits for HRCA are closed to the “gold standard" method for identification of *Mtb*, conventional culture, which allows for detection of approximately 10 to 100 organisms per milliliter of material, although theoretically may even come close to 1 bacilli [13]. Thus, the data suggested that HRCA were potential method used in clinical specimen detection. Secondly, in order to determine the specificity of HRCA, eight strains of typical NTM and three strains of MTC were analyzed. No positive bands were found in agarose gels of NTM samples in contrast to that of MTC. The data showed that HRCA is more specific for MTC, which encouraged a large-scale test of HRCA with padlock probes in clinical sputum samples.

A relatively large, controlled, and comprehensive clinical validation study was conducted in a TB specialized hospital. The data presented demonstrates a high accuracy of detection for HRCA, qRT-PCR, and culture assays, all of which were much better than smear microscopy. An exception to this general conclusion was noted in the smear-negative, culture-positive (S−/C+) group where the sensitivity of HRCA assay was statistically lower than that of qRT-PCR and culture assays. There were 3 patients with negative HRCA but positive qRT-PCR detection. Cultures from these patients presented less than 200 CFU (detection limit of HRCA) but more than 50 CFU (detection limit of in-house PCR). These data suggested that the sensitivity of the HRCA assay in smear-negative samples was inadequate for TB diagnosis.

Discordant results between HRCA, qRT-PCR, and smear assays could arise if non-tuberculosis mycobacterium (NTM) was present. In S+/C+ TB patients, two sputum specimens were smear-positive but negative by both HRCA and qRT-PCR negative. This was due to infection with M.*avium* and M.*kansasii*, confirming that HRCA and qRT-PCR were specific and able to distinguish NTM from MTC. In the control group, one potentially false positive of HRCA and qRT-PCR was found in lung cancer patients who had a history of previous TB. This suggested a sub-clinical relapse or excretion of residual persistent DNA from dead organisms, which could be positive by HRCA and qRT-PCR, but negative by culture [14].

Several commercial systems, such as the amplified Mtb direct test (Gen-probe, San Diego,CA) and Cobas TaqMan MTB PCR, have been recently developed and used for direct detection of Mtb in clinical specimens. In smear positive specimens, the sensitivity of most commercial tests ranges from 87.5% to 100% [15]. The sensitivity in the present study was 96.7%. In smear negative samples, the sensitivities of HRCA and qRT-PCR were 61.3% (46/75) and 65.3% (49/75), respectively. The sensitivity of commercial systems varies from 50.0% to 70.8%. The specificity of all experiments ranged from 98.2% to 100%. These results strongly demonstrate that HRCA and qRT-PCR can be accurately used for detection of Mtb within clinical sputum specimens.

Laboratory detection techniques are crucial tools for TB diagnosis. In recent years, nucleic acid amplification (NAA) tests have been developed and have provided a breakthrough in TB diagnosis techniques. In particular, PCR-based assays such as qRT-PCR, have been the most promising and attractive new methods to-date. These approaches offer an important and rapid tool in TB diagnosis, but they are inaccessible for low resource clinical laboratories due to the requirement for expensive laboratory infrastructure and sophisticated technical skills [16].

Compared with the routine detection tools required for qRT-PCR, HRCA has several advantages. Firstly, the procedure is very simple and does not require additional training for clinical laboratory technicians. Instruments needed for HRCA such as an incubator, water bath, etc., are standard and inexpensive for clinical laboratories. Thus, HRCA is a very cost-effective analysis. Secondly, the sensitivity and specificity of HRCA is high enough for clinical specimen analysis. An additional advantage of the HRCA identification method is that it requires only minimal amounts of starting genomic DNA. Furthermore, the sensitivity of HRCA could be improved through the fluorescence labeling of padlock probes used in the HRCA assay [17]. Thirdly, the hands-on time for performance of the entire assay is less than a day, which is significantly shorter than the time required for microbiological culture, the “gold” standard diagnostic tool for TB diagnosis. In addition, HRCA provides for clear identification of TB because positive results are simply visualized by standard agarose gel electrophoresis.

The objective of this study was the development of a simple and accessible method of TB detection for use in high-burden, low-resource and technology settings. Although the number of cases involved in this study was limited, and only sputum specimens were analyzed, our study clearly illustrated that the HRCA assay could be utilized to achieve high sensitivity and specificity in the direct detection of *Mtb*.

Currently, we are performing HRCA with fluorescence labeling padlock probes on a larger number of clinical specimens including pleural effusions and bronchoalveolar lavage fluids, amongst others. This modified approach applied to a greater variety of specimen types is being performed in order to confirm the diagnostic specificity and sensitivity of HRCA which can be reliably applied to routine laboratory diagnosis.

## References

[1] WHO (2009) Global tuberculosis control: epidemiology, strategy, financing: WHO Report 2009. WHO/HTM/TB/2009.411. WHO, Geneva, Switzerland, 1–300.

[2] Sohn H, Minion J, Albert H, Dheda K, Pai M (2009) TB diagnostic tests: how do we figure out their costs? Expert Rev Anti Infect Ther 7: 723–733. 19681700 10.1586/eri.09.52

[3] George G, Mony P, Kenneth J (2011) Comparison of the efficacies of loop-mediated isothermal amplification, fluorescence smear microscopy and culture for the diagnosis of tuberculosis. PLoS One 6: e21007. 21695047 10.1371/journal.pone.0021007 PMC3117872

[4] Nyendak MR, Lewinsohn DA, Lewinsohn DM (2009) New diagnostic methods for tuberculosis [J]. Curr Opin Infect Dwas 22: 174–182. 10.1097/qco.0b013e3283262fe9 PMC3889480 19283913

[5] Andras SC, Power JB, Cocking EC, Davey MR (2001) Strategies for signal amplification in nucleic acid detection. Mol Biotechnol 19: 29–44. 11697219 10.1385/MB:19:1:029

[6] Kaocharoen S, Wang B, Tsui KM, Trilles L, Kong F, et al (2008) Hyperbranched rolling circle amplification as a rapid and sensitive method for species identification within the Cryptococcus species complex. Electrophoresis 29: 3183–3191. 18600831 10.1002/elps.200700903

[7] WHO (1998) Laboratory services in tuberculosis control, WHO, Geneva, Switzerland. Available: http://whqlibdoc.who.int/hq/1998/WHO_TB_98.258_(part2).pdf.

[8] Young DB, Perkins MD, Duncan K, Barry CE 3rd (2008) Confronting the scientific obstacles to global control of tuberculosis. J Clin Invets 118: 1255–1265. 10.1172/JCI34614 PMC2276805 18382738

[9] Boehme CC, Nabeta P, Hillemann D, Nicol MP, Shenai S (2010) Rapid Molecular Detection of Tuberculosis and Rifampin Resistance. N Engl J Med 363: 1005–1015. 20825313 10.1056/NEJMoa0907847 PMC2947799

[10] Scherer LC, Sperhacke RD, Jarczewski C, Cafrune PI, Michelon CT, et al (2011) Comparison of two laboratory-developed PCR methods for the diagnosis of pulmonary tuberculosis in Brazilian patients with and without HIV infection. BMC Pulm Med 29 11: 15. 10.1186/1471-2466-11-15 PMC3073961 21447159

[11] Kim JH, Kim YJ, Ki CS, Kim JY, Lee NY (2011) Evaluation of Cobas TaqMan MTB PCR for detection of Mycobacterium tuberculosis. J Clin Microbiol. 49: 173–176. 10.1128/JCM.00694-10 PMC3020466 21048015

[12] Roche Molecular Systems, Inc. (2007) COBAS TaqMan MTB test. Roche Mocular Systems, Inc., Branchburg, NJ.

[13] Lighter J, Rigaud M (2009) Diagnosing childhood tuberculosis: traditional and innovative modalities. Curr Probl Pediatr Adolesc Health Care 39: 61–88. 19215860 10.1016/j.cppeds.2008.12.003

[14] Rachow A, Zumla A, Heinrich N, Rojas-Ponce G, Mtafya B, et al (2011) Rapid and accurate detection of Mycobacterium tuberculosis in sputum specimen by Cepheid Xpert MTB/RIF assay-a clinical validation study. PLoS One 6: e20458. 21738575 10.1371/journal.pone.0020458 PMC3126807

[15] Yang YC, Lu PL, Huang SC, Jenh YS, Jou R, et al (2011) Evaluation of the Cobas TaqMan MTB test for direct detection of Mycobacterium tuberculosis complex in respiratory specimens. J Clin Microbiol 49: 797–801. 21177901 10.1128/JCM.01839-10 PMC3067742

[16] Hofmann-Thiel S, Turaev L, Hoffmann H (2010) Evaluation of the hyplex TBC PCR test for detection of Mycobacterium tuberculosis complex in clinical specimen. BMC Microbiol 31 10: 95. 10.1186/1471-2180-10-95 PMC2853532 20356361

[17] Yi J, Zhang W, Zhang DY (2006) Molecular Zipper: a fluorescent probe for real-time isothermal DNA amplification. Nucleic Acids Res 34: e81. 16822854 10.1093/nar/gkl261 PMC1488881

